# In a search of a protective titer: Do we or do we not need to know?

**DOI:** 10.1002/ctm2.668

**Published:** 2021-12-12

**Authors:** Ancha Baranova, Vikas Chandhoke, Alena V. Makarova, Boris Veytsman

**Affiliations:** ^1^ School of System Biology, College of Science George Mason University Fairfax Virginia USA; ^2^ Research Center for Medical Genetics RAMS Moscow Russia; ^3^ Institute of Molecular Genetics National Research Center, Kurchatov Institute Moscow Russia

**Keywords:** antibodies, biomarkers, covid‐19, vaccine protection

## Abstract

The level of postvaccine protection depends on two factors: antibodies and T‐cell responses. While the first one is relatively easily measured, the measuring of the second one is a difficult problem. The recent studies indicate that the first one may be a good proxy for the protection, at least for SARS‐CoV‐2. The massive data currently gathered by both researcher and citizen scientists may be pivotal in confirming this observation, and the collective body of evidence is growing daily. This leads to an acceptance of IgG antibody levels as an accessible biomarker of individual's protection. With enormous and immediate need for assessing patient condition at the point of care, quantitative antibody analysis remains the most effective and efficient way to assess the protection against the disease. Let us not discount importance of reference points in the turmoil of current pandemics.

The difficulties of adequate modelling of the complex interactions between the components of the human immune system and multiple pathogens are well acknowledged. On the other hand, the clinical practice requires simple assays. This leads to the necessity of the use of biomarkers that quantitatively reflect the main features of the underlining processes without comprehensively describing them. Easily measured by a variety of relatively inexpensive immunoassays, antibodies make attractive biomarkers for the diagnostics of many diseases and for the prediction of their course. It is tempting to consider the levels of antigen‐specific antibodies in human serum as a proxy for the protection against a communicable disease, especially when talking about vaccine‐induced immunity, since the majority of modern vaccines offer protection through antibodies, due to their ability to neutralize certain antigens.^1^

Having said that, even in case of vaccines with the most indisputable role of antibodies in the preventative action, for example, the measles vaccine, there is always some space for cell‐mediated protection. As an example, in a study of comparative resistance to measles in vaccinated and unvaccinated children in rural Senagal, those who had antibody titers above certain levels were uniformly protected, regardless of the way they acquired their IgGs – through vaccine, or through transplacental transfer.^2^ At the lower antibody titers, only the vaccinated children were able to ward off the infection, while those who acquired similarly low levels from their mothers displayed a high risk of contracting measles.^2^ As the cell‐based immune components do not cross the placental barrier, the observed difference is attributable to non‐antibody driven 'invisible' hand of immune response.

Measles is the classic case of a virus that infects the mucosae at first, but then rapidly causes viremia. As antibodies accumulate in the blood, they curtail the spread of the measles virions and stop the infection. The case of the viruses largely restricted to the mucosal surfaces is substantially more complicated. For the protection against influenza, rotaviruses, papillomaviruses and many invading bacteria, antibodies must be present at the site of mucosal replication or other specific site and also must be sufficiently polyclonal to be effective against a variety of heterologous serotypes. In all these cases, there are many publications that argue the importance of CD4+ responses and other immunity biomarkers. These results are rather tricky to validate, to transfer between the labs, and finally, to translate into easily readable numbers signifying clinical acceptance.

In the case of SARS‐CoV‐2, the most recent case in point, the viremia is not a given. Recent study of Jacobs et al.^3^ demonstrated that the presence and the levels of the viral RNA in the blood, as well as the detection of the SARS‐CoV‐2 virions in pelleted plasma correlated with the severity of the disease. However, this is fairly rarely seen in outpatients (11% of the study cases) as opposed to intensive care unit (ICU) (100%). In other words, sizing up the protective immunity in SARS‐CoV‐2 may require differential approaches, specific for the selected outcome of the vaccination, that is, either the overall susceptibility to the infection or the protection against its most severe manifestations requiring the treatment in ICU.

For the sake of the following argument let us discuss only the most desired vaccination outcome ‐ the 'sterilizing' immunity which protects us from contracting SARS‐CoV‐2 disease. It can be generally described by a an equation Z = f(X,Y), where X is the antibody component of the response, Y is the complex cellular component and Z is the resultant magnitude of actual protection. In this equation, the value X (antibodies) is easily quantifiable, while the Y (the T cell responses) is evidently important, but is not readily measured. The reasons we do not use T‐cell responses as round‐the‐mill biomarker for clinical purposes are multiple. They include the fact that the 'T‐cell response' is an umbrella term rather than the specific process. They also include the obvious problem of the costs and the scalability of the cell‐based assays, especially when applied to public health in general rather than to a handful of special cases. Because of these problems at present, we collectively refuse to produce a solution for a total equation, which results in the ignorance of the actual protection due to its relative dependence on the T‐cell response. This choice of ignorance is especially bitter as the T‐cell dependence assumption may turn not critical at all, since CD8+ T cells do not account^4^ for the protection of rhesus macaques from severe COVID‐19.

To approach this problem in a most straightforward way, we could conduct an enormous clinical trial with, say, a million of participants, who will go through thorough examination at the beginning, then receive a vaccine and subject themselves to multiple samplings of blood which will be assayed both for the levels of antibodies and for activation of various T cells with a variety of SARS‐CoV‐2 antigens. All these samplings and tests should be performed in standardized conditions, multiple times till the trial participants would naturally contract the disease. Even better, if we carefully measure the titer of the virus itself, and then infect volunteers. It is obvious that the brute‐force trials of this kind are completely impossible.

If the function f(X,Y) is arbitrary, the problem is unsolvable. Fortunately, it is not arbitrary. If we have some idea about the mechanism of protection, we can make reasonable assumptions about this function: we can create a plausible model and work with it. For example, we may note that the production of antibodies is correlated with the amounts of T cells of specific subtype, or with the degree of their activation. Thus if we assume that X and Y are correlated, we can use the measured level X for the prediction of Z. Indeed, the individual magnitudes of T‐cell response to the spike, as measured by interferon‐gamma (IFN‐γ) release assay are significantly associated with the neutralizing potency of antibodies from same individuals, as measured by Zollner et al.^5^ using SARS‐CoV‐2 microneutralization test. Similar findings were made by Zuo et al.^6^ The work of Liu et al.,^7^ which is presented in current issue of CTM, provides us with another shortcut to an approximation of Z by showing an overall positive correlation of serum levels of IgG and ID50 titers for both the standard SARS‐CoV‐2 and its variants. These ideas are in agreement with a recent mathematical model for SARS‐CoV‐2 immune protection^8^ suggesting the neutralizing antibody levels are highly predictive of immune protection from symptomatic SARS‐CoV‐2 infection, and the recent UK population‐based study,^9^ which identified the thresholds for the antibody levels protecting against SARS‐CoV‐2 alpha strain. These examples are not isolated, as data are constantly trickling in, being sourced from both populations and hospital‐specific cohorts.

In many cases, the data are readily available. Researchers may access data collected by citizen science initiatives led by self‐organized groups in countries where standardized tests for blood antibody levels are accessible for consumers for reasonable prices. For example, a Russian Telegram self‐research group has collected the information about the levels of IgG on standardized assays Abbott Architect SARS‐CoV‐2 IgG RBD II and LIAISON SARS‐CoV‐2 S1/S2 IgG from 967 residents of Moscow and St. Petersburg and the consequent self‐reported polymerase chain reaction (PCR)‐positive cases of COVID‐19 within the frame of 2 months.^10^ Among 49 cases, 91.7% people had the IgG levels <370 BAU/ml (Abbott II) and <252 AU/ml (LIASON).^11^These data can be a treasure trove for acquired immunity research.

It is important to acknowledge, however, that the distribution of efforts often leads to the bias in the collected results. More structured and controlled studies are warranted to affirm results obtained thus far. Lack of specificity and technical limitations of precisely determining cell‐based immune response makes them unfeasible as a clinical measure. With enormous and immediate need for assessing patient condition at the point of care, quantitative antibody analysis remains the most effective and efficient way to assess the protection against the disease. Let us not discount importance of reference points in the turmoil of current pandemics.

## CONFLICT OF INTEREST

There are no competing interests to report.
